# 

**Published:** 1849-04

**Authors:** 


### CONTENTS

OF NO. IV.

### ORIGINAL COMMUNICATIONS.

### ESSAYS AND CASES.

ART. I.—Thoughts on the Origin of Moles (Germen Falsum), and on certain properties of the Secale Cornutum, illustrated by Cases. By Dr. THOS. LIPSCOMB, of Shelbyville, Tenn. ....................277

ART. II.—A Case of Uterine Hemorrhage following the expulsion of the Placenta. By L. P. YANDELL, M.D. .....287

ART. III.—A Case of Moral Insanity. By B. M. WIBLE, M.D., of Louisville, Ky. ....................289

### REVIEWS.

ART. IV.—1. The British and Foreign Medico-Chirurgical Review. 2. The London Lancet, for January and February. 3. The true Pathological Nature of Cholera, and an Infallible Method of treating it. By GEO. STUART HAWTHORNE, M.D. 4. On the Cryptogamous Origin of Malarious and Epidemic Fevers. By J. K. MITCHELL, A.M., M.D., Professor of Practical Medicine in Jefferson Medical College....................303

ART. V.—An Illustrated System of Human Anatomy, Special, General, and Microscopic. By SAMUEL GEO. MORTON, M.D., Penn. and Edinb., Member of the Medical Societies of Philadelphia, New York, Boston, Edinburgh, and Stockholm ....................332

SELECTIONS FROM AMERICAN AND FOREIGN JOURNALS.

Synopsis of the Methods of Treating Asiatic Cholera ..........337
Chloroform in various diseases..........................340
On the Utility of Trisnitrate of Bismuth in the Diarrhea accompanying Phthisis ..........343
Quinine as a Prophylactic in Puerperal Fever ..........344
Sulphate of Quinine in Fever ..........344
Ship Fever..........347
Case of Traumatic Tetanus..........357

ORIGINAL INTELLIGENCE.

Editorial Changes ..........361
Commencement in the University of Louisville ..........361
Cholera ..........362
Cholera in New Orleans ..........363
The Cholera ..........363
Louisville Marine Hospital..........364
Transactions College of Physicians..........365
Medical School Changes ..........365
Boston Medical and Surgical Journal ..........366
Anecdote of M. Orfila ..........366
Medical Association..........368
To Correspondents and Publishers..........368

